# Emissions of ammonia and hydrogen sulfide from typical dairy barns in central China and major factors influencing the emissions

**DOI:** 10.1038/s41598-019-50269-y

**Published:** 2019-09-25

**Authors:** Zhifang Shi, Xiaoqin Sun, Yao Lu, Lei Xi, Xin Zhao

**Affiliations:** 1 College of Animal Science and Technology, Northwest A&F University, Yangling, Shaanxi 712100 China; 2 College of Animal Science and Technology, Henan University of Animal Husbandry and Economy, Zhengzhou, Henan 450046 China; 3 Department of Animal Science, McGill University, 21,111 Lakeshore, Ste. Anne de Bellevue, Quebec H9X 3V9 Canada

**Keywords:** Agroecology, Environmental impact, Environmental impact

## Abstract

There are few studies on the concentrations and emission characteristics of ammonia (NH_3_) and hydrogen sulfide (H_2_S) from Chinese dairy farms. The purpose of this study was to calculate the emission rates of NH_3_ and H_2_S during summer and to investigate influencing factors for NH_3_ and H_2_S emissions from typical dairy barns in central China. Eleven dairy barns with open walls and double-slope bell tower roofs from three dairy farms were studied. Five different locations in each barn were sampled both near the floor and at 1.5 m above the floor. Concentrations of NH_3_ and H_2_S were measured using the Nessler’s reagent spectrophotometry method and the methylene blue spectrophotometric method, respectively. NH_3_ concentrations varied between 0.58 and 4.76 mg/m^3^ with the average of 1.54 mg/m^3^, while H_2_S concentrations ranged from 0.024 to 0.151 mg/m^3^ with the average of 0.092 mg/m^3^. The concentrations of NH_3_ and H_2_S were higher during the day than at night, and were higher near the ground than at the height of 1.5 m, and were higher in the manure area than in other areas. NH_3_ and H_2_S concentrations in the barns were significantly correlated with nitrogen and sulfur contents in feed and manure (*P* < 0.05), and with temperature inside the barns (*P* < 0.05). Calculated emission rates of NH_3_ ranged from 13.8 to 41.3 g NH_3_/(AU·d), while calculated emission rates of H_2_S ranged from 0.15 to 0.46 g H_2_S/(AU·d). These results will serve as a starting point for a national inventory of NH_3_ and H_2_S for the Chinese dairy industry.

## Introduction

Global production and consumption of animal products will continue to expand^1^. However, animal production has been linked to a number of contentious environmental issues in recent decades, including soil erosion, production of global greenhouse gases and atmospheric pollution^2,3^. For example, emission of ammonia (NH_3_) and hydrogen sulfide (H_2_S) from livestock production contributes to atmospheric pollution^4,5^. Thus, this has to be addressed from regulatory and environmental standpoints. Livestock production contributes 64% of total anthropogenic NH_3_ emissions on a global scale^4^. The exact number for the contribution of H_2_S from livestock production in China or in the world is not available. In Denmark, H_2_S from agricultural sources becomes a more significant fraction for the total sulfur emissions to the atmosphere, as power generation and combustions become cleaner and emit less sulfur dioxide (SO_2_)^5^. Among different livestock species, contribution of dairy farming to NH_3_ and H_2_S emission is significant. Dairy farming contributed about 50% of the total NH_3_ emission in the Netherlands^6^. China has become the world’s largest source of ammonia (NH_3_) emissions from livestock production (about 7.3 Teragram per year) and of this, 7% of the emissions could be from dairy cows^7^. In 2016, China had 12.72 million of dairy cows, ranking the third after India and Brazil in the world^8^. With the increasing demand of milk products per capita and continuous expanding of dairy farming in China, the emission of NH_3_ and H_2_S caused by dairy farming is expected to further increase. Henan province, located in the central China, is one of the five major dairy producing areas and one of air heavily polluted regions in China^9^. The amounts of NH_3_ and H_2_S produced and released from dairy farms could be large. Therefore, it is necessary to accurately estimate the NH_3_ and H_2_S produced by the dairy farming in this region to assess its impact on the environment pollution.

Gaseous NH_3_ and H_2_S from animal production come from decomposition of nitrogen- and sulfur- containing compounds in excrement. NH_3_ is an important odor gas in the livestock barns as well as an important neurotoxic substance^10^. Emitted H_2_S is formed by anaerobic degradation of sulphur-containing organic compounds, especially proteins^11^. Hydrogen sulfide is a prominent gaseous constituent in animal buildings and manure storage^12^. It has been considered as the most dangerous gas from livestock production systems and it is responsible for deaths of animals and farm workers in animal facilities^13,14^. Chronic exposure to H_2_S can lead to respiratory diseases, eye diseases, and neurological diseases^15^. With a density higher than air, H_2_S tends to accumulate in the poorly ventilated areas which exacerbates its hazardous impact. Emission of H_2_S also contributes to the atmospheric burden of sulfur compounds, which have a major role in the formation of secondary aerosols through oxidation and conversion to aerosol sulfate^16,17^.

Generation of NH_3_ and H_2_S in dairy barns is affected by several factors such as manure production and storage, manure disturbance, ambient temperature, and air exchange rate^18–20^. The concentration of gases inside barns is especially affected by a ventilation system and building structures, which are usually designed to regulate room temperature, especially during summer. Emissions of NH_3_ and H_2_S from dairy farms in China may significantly differ from those in other regions such as Europe and the United States due to differences in climatic conditions, feeding methods, rations and configuration of dairy barns. Except in the Northeastern region, dairy barns in most parts of China have bell tower roofs and rely on wind pressure or thermal buoyancy for natural ventilation. Axial fans and sprinkler systems are also installed to help reducing the heat load during summer. Atmospheric NH_3_ and H_2_S from animal production have negative effects on health of animals and humans and the ecosystem^21^. From a policy perspective, governments need to have accurate estimates of emissions and fate of NH_3_ and H_2_S in their jurisdictions. Due to the technique difficulty and high expense of field studies, most reported emission rates of NH_3_ and H_2_S were calculated from models. There was only one field study for NH_3_ emissions from dairy farms in the Northern China^22^. While the inverse dispersion technique in combination with an open-path tunable diode laser used in that study^22^ is sensitive and fast, it is highly dependent on the meteorological conditions with big variations of determined values in addition to the need of the special equipment. The chemical methods used in this study provide higher accuracy of the gas concentrations. However, they are more time-consuming and labor-intensive. There are no reported measurements of H_2_S emissions from Chinese dairy farms. As a first tempt to provide accurate and reliable estimation of NH_3_ and H_2_S emissions from Chinese dairy farms, this study aimed to understand emission patterns of NH_3_ and H_2_S in typical open barns during summer in central China, in order to provide the basis for emission reduction and regulation of NH_3_ and H_2_S for the dairy industry in China.

## Results

### Environmental parameters

**Figure 1:**
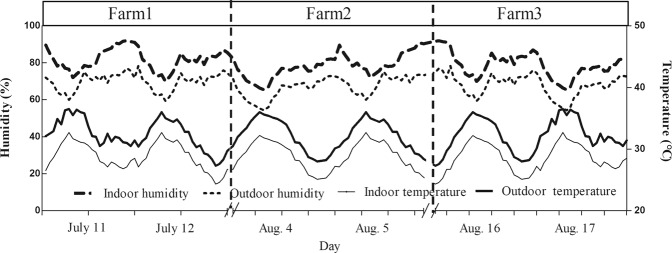
Diel changes of indoor and outdoor temperature and humidity. For each barn, measurement was performed at 2 outside locations (2 upwind blank areas 20 m away from the barn) and 5 inside locations (2 cow bed locations, 2 manure areas and 1 feeding alley) as indicated in Fig. 6. Each location was sampled both near the floor and at 1.5 m above the floor. Measurements were made every 2 hours for two days. The values for temperature and humidity in the figure represent the average of the values measured near the floor and at 1.5 m above the floor.

As shown in Table 1, the average of indoor temperature was significantly lower than the average of outdoor temperature by 5.3 °C. On the other hand, the relative humidity, the average of wind speed and CO_2_ concentrations were significantly higher inside the barn than outdoor by 14.7%, 47.2% and 24.4%, respectively. There was no significant difference of the average of air pressure and total suspended particles (TSP) inside or outside of the dairy barns. Diel changes of temperature and humidity for indoor and outdoor were shown in Fig. 1. During the experimental periods, the indoor temperature was lower than the outdoor temperature, while the indoor humidity was higher than the outdoor humidity.

**Table 1:** The averages of environmental parameters inside and outside of dairy barns.

Parameters	Indoor					Outdoor				
	Means	SD	CV (%)	Min	Max	Mean_2_	SD	CV (%)	Min	Max
Temperature (°C)	27.8^b^	2.7	9.71	26.8	32.7	33.1^a^	3.7	11.18	30.5	38.7
Humidity (%)	80.6^a^	9.3	11.68	65.0	99.5	70.3^b^	10.1	14.37	55.0	89.5
Wind speed (m/s)	1.81^a^	0.28	15.47	1.13	2.19	1.23^b^	0.38	30.89	0.63	2.69
CO_2_ (mg/m^3^)	534.1^a^	108.6	11.63	739.9	1718	429.3^b^	112.7	13.59	384.4	512.7
Air pressure (k pa)	99.2	1.2	1.21	89.6	99.8	99.2	1.2	1.21	89.6	99.8
TSP (mg/m^3^)	0.262	0.027	10.31	0.226	0.304	0.232	0.023	11.39	0.188	0.256

For each barn, environmental parameters were measured at 2 outside locations (2 upwind blank areas 20 m away from the barn) and 5 inside locations (2 cow bed locations, 2 manure areas and 1 feeding alley) as indicated in Fig. 6. Each location was sampled both near the floor and at 1.5 m above the floor. Measurements were made every 2 hours for 48 hours. Means in the table were the average of all values at 2 heights during the experiment. ^a,b^Means with different letters within the same row are significantly different (*P* < 0.05).

### Concentrations and diel changes of NH_3_ and H_2_S

As shown in Table 2, the average of indoor NH_3_ concentration (1.54 mg/m^3^) for 11 dairy barns was 60.4% higher than that outside (0.96 mg/m^3^) of the barns (*P* < 0.05) during the 48 hours of measurement. In addition, the average NH_3_ concentration inside the 6 lactating barns (2.13 mg/m^3^) was 156.6% higher than that inside the 5 non-lactating barns (0.83 mg/m^3^) (*P* < 0.05). Similarly, the average of the indoor H_2_S concentration (0.092 mg/m^3^) for 11 dairy barns was 240.7% higher than that outside (0.027 mg/m^3^) of the barns (*P* < 0.05) (Table 3). The average H_2_S concentration inside the 6 lactating barns (0.125 mg/m^3^) was 140.4% higher than that inside the 5 non-lactating barns (0.052 mg/m^3^) (*P* < 0.05) (Table 3).

**Table 2:** NH_3_ concentrations (mg/m^3^) inside and outside of each dairy barn.

Cow barns	Indoor					Outdoor				
	Mean	SD	CV (%)	Min	Max	Mean^2^	SD	CV (%)	Min	Max
M1	4.76	3.26	68.49	0.49	10.98	1.34	0.83	61.94	0.03	2.29
M2	1.68	1.22	72.62	0.13	5.69	1.26	0.75	59.52	0.05	1.43
M3	1.59	1.05	66.04	0.01	5.39	1.39	0.82	58.99	0.06	2.18
M4	2.03	1.52	74.88	0.19	6.23	1.00	0.57	57.00	0.04	1.45
M5	1.59	1.14	71.70	0.27	3.78	1.25	0.80	64.00	0.05	1.45
M6	1.11	0.84	75.68	0.15	4.05	0.88	0.61	69.32	0.03	1.22
N1	0.58	0.38	65.52	0.02	1.45	0.47	0.33	70.21	0.03	1.21
N2	0.89	0.46	51.69	0.11	1.69	0.69	0.33	47.83	0.02	1.13
N3	1.08	0.92	85.19	0.21	3.93	1.00	0.57	57.00	0.02	2.21
N4	0.49	0.22	44.90	0.18	1.85	0.31	0.20	64.52	0.01	1.11
N5	1.10	0.89	80.91	0.41	3.99	1.01	0.43	42.57	0.02	2.22
**Mean(M)**	**2.13** ^**a**^	**1.51**	**70.89**	**0.21**	**6.02**	**1.19** ^**a**^	**0.73**	**61.34**	**0.04**	**1.67**
**Mean(N)**	**0.83** ^**b**^	**0.57**	**68.67**	**0.19**	**2.58**	**0.70** ^**b**^	**0.37**	**52.86**	**0.02**	**1.58**
**Mean(T)**	**1.54**	**1.08**	**70.13**	**0.20**	**4.46**	**0.96**	**0.57**	**59.38**	**0.03**	**1.63**

For each barn, NH_3_ concentrations were measured at 2 outside locations (2 upwind blank areas 20 m away from the barn) and 5 inside locations (2 cow bed locations, 2 manure areas and 1 feeding alley) as indicated in Fig. 6. Each location was sampled both near the floor and at 1.5 m above the floor. Measurements were made every 2 hours for 48 hours. Letter M means a lactating cow barn, while letter N indicates a non-lactating cow barn. Means in the table were the average of all values during the experiment. Mean (M) is the average of 6 lactating barns (M1 to M6). Mean (N) is the average of 5 non-lactating barns (N1to N5). Mean (T) is the average of all 11 cow barns. ^a,b^Means with different letters within the same column are significantly different (*P* < 0.05).

**Table 3:** H_2_S concentrations (mg/m^3^) inside and outside of each dairy barn.

Cow barns	Inside					Outside				
	Mean	SD	CV (%)	Min	Max	Mean^2^	SD	CV (%)	Min	Max
M1	0.112	0.051	45.54	0.027	0.227	0.088	0.065	73.86	0.038	0.132
M2	0.128	0.047	36.72	0.034	0.156	0.035	0.014	40.00	0.044	0.053
M3	0.131	0.051	38.93	0.044	0.207	0.029	0.021	72.41	0.000	0.055
M4	0.132	0.072	54.55	0.073	0.195	0.047	0.019	40.43	0.002	0.076
M5	0.098	0.037	37.76	0.067	0.156	0.024	0.017	70.83	0.005	0.044
M6	0.151	0.063	41.72	0.091	0.177	0.022	0.011	50.00	0.000	0.055
N1	0.024	0.013	54.17	0.012	0.048	0.014	0.002	14.29	0.011	0.027
N2	0.066	0.048	72.73	0.023	0.137	0.008	0.005	62.50	0.000	0.014
N3	0.039	0.027	69.23	0.015	0.046	0.017	0.005	29.41	0.013	0.019
N4	0.056	0.021	37.50	0.041	0.075	0.013	0.01	76.92	0.001	0.024
N5	0.075	0.053	70.67	0.027	0.112	0.005	0.003	60.00	0.001	0.031
**Mean(M)**	**0.125** ^**a**^	**0.054**	**42.53**	**0.056**	**0.186**	**0.041** ^**a**^	**0.025**	**57.92**	**0.015**	**0.069**
**Mean(N)**	**0.052** ^**b**^	**0.032**	**60.86**	**0.024**	**0.084**	**0.011** ^**b**^	**0.005**	**48.62**	**0.005**	**0.023**
**Mean(T)**	**0.092**	**0.044**	**50.87**	**0.041**	**0.140**	**0.027**	**0.016**	**53.70**	**0.010**	**0.048**

For each barn, H_2_S concentrations were measured at 2 outside locations (2 upwind blank areas 20 m away from the barn) and 5 inside locations (2 cow bed locations, 2 manure areas and 1 feeding alley) as indicated in Fig. 6. Each location was sampled both near the floor and at 1.5 m above the floor. Measurements were made every 2 hours for 48 hours. Letter M means a lactating cow barn, while letter N indicates a non-lactating cow barn. Means in the table were the average of all values during the experiment. Mean (M) is the average of 6 lactating barns (M1 to M6). Mean (N) is the average of 5 non-lactating barns (N1to N5). Mean (T) is the average of all 11 cow barns. ^a,b^Means with different letters within the same column are significantly different (*P* < 0.05).

**Figure 2:**
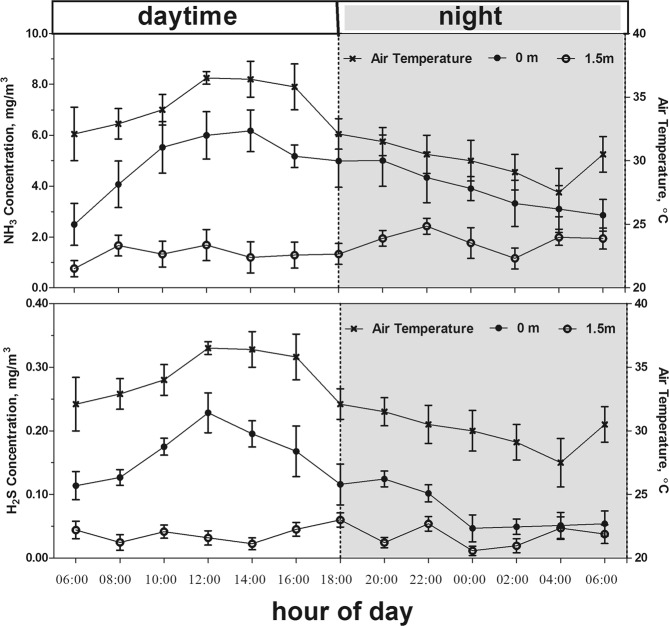
Diel changes of NH_3_ and H_2_S concentrations as well as the temperature inside the dairy barns. For each barn, measurement of NH_3_ and H_2_S was performed at 2 outside locations (2 upwind blank areas 20 m away from the barn) and 5 inside locations (2 cow bed locations, 2 manure channel locations and 1 feeding alley location) as indicated in Fig. 6. Each location was sampled both near the floor and at 1.5 m above the floor. Measurements were made every 2 hours for 48 hours. The values for NH_3_ and H_2_S represent the average of each sampling time point during a day for each height. The values for indoor temperature represent the average of the values measured both near the floor and 1.5 m above the floor.

Figure 2 shows the diel changes of NH_3_ and H_2_S concentrations in lactating barns measured both near the floor and at 1.5 m above the floor as well as the diel change of the temperature inside the barns at 1.5 m above the floor. The concentrations of NH_3_ and H_2_S during daytime were higher than those during night. The concentrations of NH_3_ and H_2_S near the floor were higher than those measured at the height of 1.5 m above the floor. In addition, the diel changes of NH_3_ and H_2_S concentrations near the floor were parallel to the diel change of the temperature inside the barns. There were no significant diurnal variations in NH_3_ and H_2_S concentrations measured at 1.5 m above the floor (*P* > 0.05), possibly due to lower concentrations and the stable wind from the axial flow fans.

**Figure 3:**
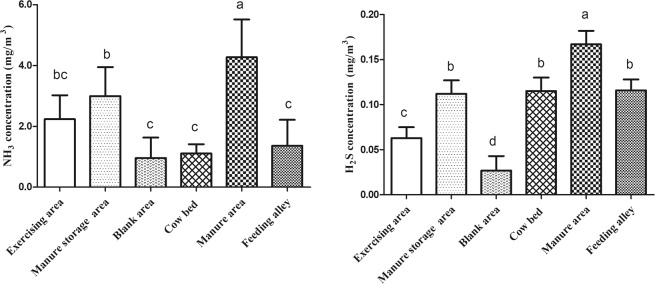
Comparison of NH_3_ and H_2_S concentrations at different locations inside and outside of dairy barns. Measurement was performed at 36 outside locations (11 exercising areas, 3 manure storage areas and 22 upwind blank areas 20 m away from the barn) and 55 inside locations (22 cow bed locations, 22 manure areas and 11 feeding alley). Each location was sampled both near the floor and at 1.5 m above the floor. Measurements were made every 2 hours for 48 hours. The NH_3_ or H_2_S concentrations were the average of the values measured both at 0 m and 1.5 m during 48 hours. The different letters within the same panel are significantly different (*P* < 0.05).

As shown in Fig. 3, there were significant differences in NH_3_ and H_2_S concentrations among different locations of the dairy farm. The average NH_3_ concentration in the manure area was 4.27 ± 1.25 mg/m^3^, which was significantly higher than those for all other areas (*P* < 0.05). The average NH_3_ concentrations among the blank area (0.96 ± 0.67 mg/m^3^), cow bed (1.11 ± 0.31 mg/m^3^), and feeding alley locations (1.36 ± 0.86 mg/m^3^) were not significantly different and were significantly lower than those at manure area and manure storage area (*P* < 0.05). Similarly, the H_2_S concentration at the manure area (0.167 ± 0.015 mg/m^3^) was the highest and significantly higher than those from other locations (*P* < 0.05). The lowest concentration of H_2_S was found in the blank area, being only 0.027 ± 0.016 mg/m^3^, which was significantly lower than those in the other areas (*P* < 0.05). There was no significant difference (*P* > 0.05) of H_2_S among the manure storage area (0.112 ± 0.015 mg/m^3^), cow bed (0.115 ± 0.013 mg/m^3^), and feeding alley (0.116 ± 0.012 mg/m^3^).

### Influence of feed N and S contents and environment parameters on NH_3_ and H_2_S concentrations

Emissions of NH_3_ and H_2_S in the dairy barns are often related to the nutrition level of the feed, the environmental factors and manure management. This study investigated relationships between the concentrations of NH_3_ and H_2_S and the following factors: the N content in the feed (*N*_*f*_), N content in manure (*N*_*m*_), N content in the urine (*N*_*u*_), S content in the feed (*S*_*f*_), S content in manure (*S*_*m*_), S content in urine (*S*_*u*_), indoor temperature (*T*_*in*_), indoor wind speed *W, CO*_2_ and *TSP* concentrations.

According to the Pearson correlation analysis, the coefficient r values with indoor NH_3_ concentrations for *N*_*f*_*, N*_*m*_*, N*_*u*_, and *T*_*in*_ were 0.912, 0.884, 0.844 and 0.781, respectively and all the correlations were highly significant (*P* < 0.01). Similarly, the correlation between indoor H_2_S concentrations and *S*_*f*_, *S*_*m*_, *S*_*u*_, and *T*_*in*_ were significant (*P* < 0.01) and their r values were 0.959, 0.961, 0.949 and 0.857, respectively. On the other hand, the correlation between indoor NH_3_ or H_2_S concentrations and *W*, *CO*_2_ and *TSP* were not significant (*P* > 0.05). Consequently, these variables (*W*, *CO*_2_ and *TSP*) were excluded for further modeling analyses.

A stepwise regression method was used to eliminate the influence of the multi-collinearity of independent variables on the accuracy of the model. Consequently, the regression models were obtained as Eqs 1 and 2 and such models were proved reliable through both the F test and the Durbin Watson test.

1 $${C}_{{{\rm{NH}}}_{{\rm{3}}}}=-\,13.877+6.637{N}_{f}+0.266\,{T}_{in}$$

2 $${C}_{{H}_{2}S}=-\,0.193+7.494{E}^{-5}{S}_{f}+0.005\,{T}_{in}$$

*C*_NH__3_ and *C*_H__2__S_ stand for the concentration of NH_3_ or H_2_S, respectively.

### NH_3_ and H_2_S emission rates

The emission rates of NH_3_ and H_2_S were determined by both CO_2_-Balance method and the wind pressure and temperature difference forces method (WT method). The animal unit (AU) is defined as a 500 kg dairy cow. As shown in Table 4, the emission rates of both NH_3_ and H_2_S in lactating barns were higher than those in non-lactating barns. The NH_3_ emission rate in the lactating barns was higher than that in non-lactating barns by 30.4% according to the CO_2_-balance method. Similarly, the H_2_S emission rate in the lactating barns was higher than that in non-lactating barns by 18.4% according to the CO_2_-balance method.

**Table 4:** Emission rates of NH_3_ and H_2_S for the CO_2_ balance method and the wind pressure and temperature difference forces (WT) method.

Gas	type	CO_2_-balance		WT method	
		range	mean	range	mean
NH_3_ g NH_3_/(AU·d)	Lactating cow barn	20.4–41.3	30.6	28.1–62.2	42.7
	Non-lactating cow barn	13.8–33.3	22.9	10.7–57.2	38.0
H_2_S g H_2_S/(AU·d)	Lactating cow barn	0.20–0.46	0.28	0.26–0.58	0.37
	Non-lactating cow barn	0.15–0.28	0.24	0.19–0.39	0.25

## Discussion

### NH_3_ and H_2_S concentrations

**Figure 4:**
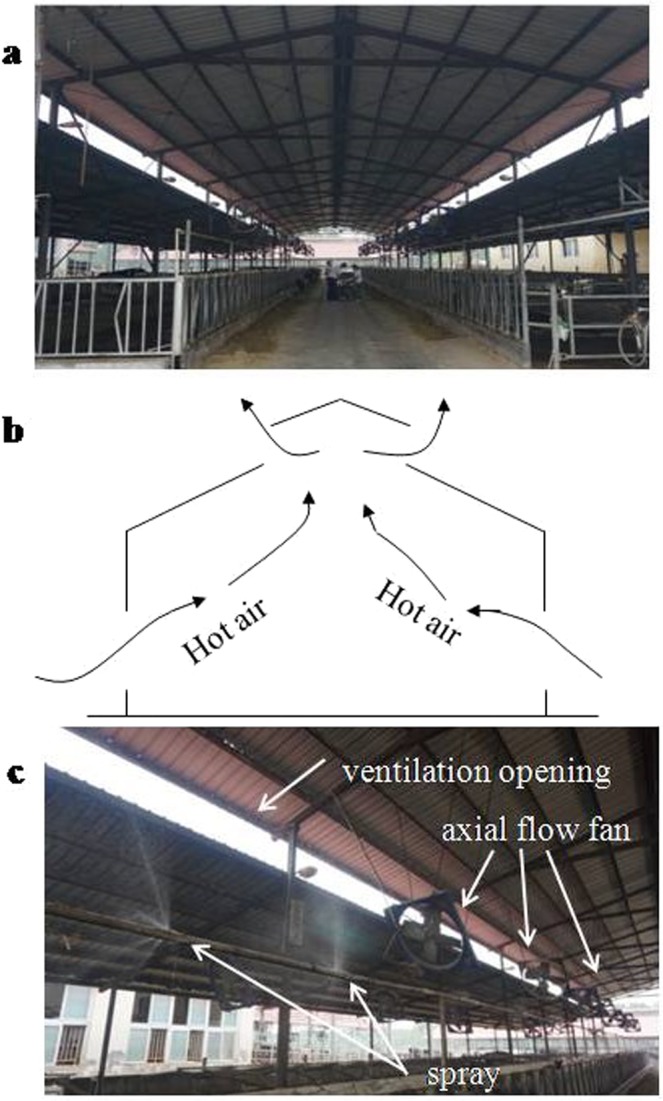
Cow barns. (**a**) A picture of a typical dairy barn; (**b**) The diagram of ventilation through a bell tower shaped roof; (**c**) Axial fans and the sprinkler cooling system. Each barn has several axial fans installed 3 m above the floor, while the spraying device was installed 2 m above the floor, which could spray water droplets of 10 μm in diameter.

Our research showed that the NH_3_ concentrations from our dairy barns during summer ranged from 0.58 to 4.76 mg/m^3^ with an average of 1.54 mg/m^3^. The average of 6 lactating barns was 2.13 mg/m^3^ and the average of 5 non-lactating barns was 0.83 mg/m^3^. The results were significantly lower than those reported by Maasikmets *et al*.^18^ (8.10–19.94 mg/m^3^) in Estonia and slightly lower than those reported by Ngwabie *et al*.^19,23^ (2.43 ± 0.99 mg/m^3^ and 3.11 ± 0.83 mg/m^3^) in the South of Sweden. Good ventilation from the natural ventilation assisted with axial fans and the spray cooling system could be the reason for the lower NH_3_ concentrations in our study. The unique barn structure with no walls and a double-slope bell tower shaped roof increases the air flow in the barn, and accelerates the gas exchange with the outside of the barn. The airflow inside the barn could create a relatively negative pressure environment inside the barn, as shown in Fig. 4. The fans accelerated the airflow. At the same time, the small water droplet from the sprinklers could dissolve a certain amount of NH_3_ and H_2_S and thus reduce the NH_3_ and H_2_S concentrations inside the barn.

The indoor H_2_S concentrations ranged from 0.024 to 0.151 mg/m^3^ with an average of 0.092 mg/m^3^. The average of 6 lactating barns was 0.125 mg/m^3^ and the average of 5 non-lactating barns was 0.052 mg/m^3^ in this study. Our indoor H_2_S concentrations were similar to those reported by Maasikmets *et al*.^18^ (0.090–0.188 mg/m^3^) and Clark *et al*.^24^ (0.145 mg/m^3^), due to good ventilation as discussed above. With a density higher than air, H_2_S tends to accumulate in poorly ventilated areas. H_2_S is only emitted when manure is disturbed through different handling processes^25^. Incidences of death caused by H_2_S have been reported for both animals^12,26^ and humans^14,27^. These deaths all happened during mixing of manure. Although the concentration of H_2_S in this study is not high, the chronic toxicity and the acute toxicity caused by disturbance of manure should not be ignored.

### Spatiotemporal characteristics of NH_3_ and H_2_S concentrations

The daytime NH_3_ and H_2_S concentrations in dairy barns were higher than those during the night in the current study (Fig. 2). Our results are in line with the findings by Wu *et al*.^28^ regarding NH_3_ emissions from naturally ventilated barns in Denmark. In addition, the diel changes of NH_3_ and H_2_S concentrations near the floor were parallel to the diel change of the temperature inside the barn (Fig. 2). The NH_3_ formation and release are affected by the temperature^29^. The temperature affects the urease activity in the excrement and a higher temperature increases the urease activity, accelerating urea decomposition into NH_3_. Formation and release of H_2_S from manure are also expected to be affected by the temperature, since the decomposition of S-containing organic compounds in manure is enzyme-dependent^30^.

The concentrations of NH_3_ and H_2_S near the floor were higher than those measured at 1.5 m above the floor in the dairy barns. In addition, the concentrations of NH_3_ and H_2_S were the highest near the manure aisle among different sampling locations (Fig. 3). Both NH_3_ and H_2_S come from the decomposition of N and S containing substances in manure and urine. The airflow pattern within the barns diluted NH_3_ and H_2_S in the air and was also responsible for the lower NH_3_ and H_2_S concentrations at 1.5 m above the floor. The study by Saha *et al*.^20^ also confirmed that the NH_3_ concentration and its discharge rate in naturally ventilated barns were affected by wind speed. The height of 1.5 m could be considered the height of the breathing line for cows and humans. The reduced concentrations of NH_3_ and H_2_S at this level can reduce the negative effects of these gases on dairy cows and farmers.

### N and S contents in feed and the concentrations of NH_3_ and H_2_S

Multivariate linear regression analyses showed that the concentrations of NH_3_ and H_2_S in the dairy barns were closely related to the contents of N and S in the feed, in addition to the significant influence by the temperature inside the barns. Concentrations of NH_3_ and H_2_S were significantly higher in lactating barns than those in non-lactating dairy barns. Lactating cows had a higher feed intake and their feed nutrient concentrations were much higher than non-lactating dairy cows. A significant correlation between the S content in the feed of dairy cows and the level of H_2_S in the air of the barn has been reported before^25^. Similarly, a higher level of dietary protein levels resulted in higher concentrations of total nitrogen in fresh manure and urine^31^. The decomposition of urea leads to the rapid rise of NH_3_ concentration in the barns.

### Emission rates

Typically, emission estimates are calculated using emission factors (EF) and numbers of animals^32^. The available emission rates for NH_3_ and H_2_S in the literature mainly come from European and American countries, including Estonia^18^, Germany^20,29^, Denmark^28^, Switzerland^33,34^, Portugal^35^, and the United States^36^.

Quantification of emissions from naturally ventilated buildings has been a complicated and challenging task, as a result of difficult and inaccurate determination of airflow rates. Several methods have been developed, each with its own advantages and drawbacks^37,38^. Among them, the CO_2_ mass balance method and the pressure difference method have been used for naturally ventilated buildings for cattle. Carbon dioxide formed by animal respiration can be used as a natural tracer gas, assuming that CO_2_ can be mixed very well with the air inside the building. However, the molecular weight of CO_2_ is 44.01 and is higher than the average molecular weight of air (28.96), making CO_2_ accumulating near the floor surface and the CO_2_ balance method somewhat flawed. Additional drawback for the CO_2_ mass balance method is inaccurate estimation of the CO_2_ production due to variations by animals^37^. The ventilation rate throughout a naturally ventilated barn is dependent on both thermal buoyancy forces and wind pressure on the openings of the building. Thus, the wind pressure and temperature difference forces method calculates the ventilation rate through determination of wind speed and temperature inside the barn. The main drawbacks of this approach are the non-uniform distribution of the pressure differences and the velocity profile across the ventilation openings and through time, especially for barns with very large openings. This method tends to overestimate the emission rates^38^, which was also seen in the current study (Table 4). Therefore, while the results calculated from both methods are presented for the purpose of comparison, we prefer to use the results from the CO_2_ method.

**Figure 5:**
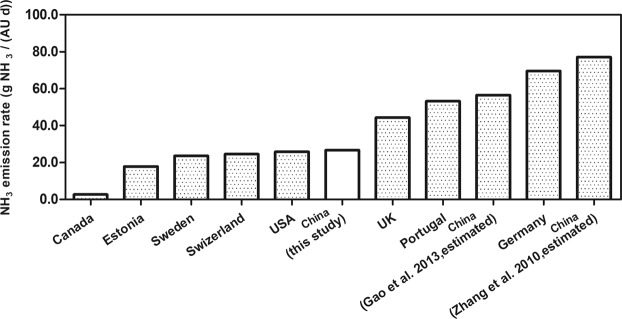
Comparison of ammonia emission rates between this study and those reported in the literature.

The average NH_3_ emission rate was 30.6 g NH_3_/(AU·d), while the average H_2_S emission rate was 0.28 g H_2_S/(AU·d) for lactating cow barns. On the other hand, the average NH_3_ emission rate was 22.9 g NH_3_/(AU·d) and the average H_2_S emission rate was 0.24 g H_2_S/(AU·d) in non-lactating cow barn for this study, based on the CO_2_ balance method. The higher emission rates of NH_3_ and H_2_S for the lactating cow barns than those for the non-lactating cow barns may be related to the higher content of nitrogen and sulfur in the feed for lactating cows. As shown in Fig. 5, our emission rate for NH_3_ (26.75 g NH_3_/AU·d) was slightly higher than those reported for Estonia^18^, Sweden^19^, and the USA^39^. On the contrary, our number was slightly lower than those reported for the UK^40^, Portugal^35^, and Germany^41^. Interestingly, our NH_3_ emission rate was much lower than those previously estimated for Chinese dairy farms^42,43^. The average emission rate of H_2_S for all dairy barns was 0.26 g H_2_S/(AU·d), which is much higher than that reported by Maasikmets *et al*.^18^ (0.14 ± 0.08gH_2_S/(AU·d)) of the Estonia. Multiple factors such as the difference of measurement methods, feeding levels, environmental effects and barn structures could be responsible for different emission rates of different countries. The major difference of the emission rates of NH_3_ between the current study and the previous studies for China calls for more field studies.

## Materials and Methods

### Dairy farms and herd profile

The study was conducted from July 4 to August 21, 2017 inside and outside of 11 dairy barns in three farms in Henan province. Henan province is located in the central China, with east longitude 110°21′~116°39′ and north latitude 31°23′~36°22′. The farms are more than 1 km away from residential areas. The detailed description of these dairy barns is in Table 5. All 11 barns are naturally ventilated through open walls and a double-slope bell tower shaped roof (Fig. 4). Additionally, 32 axial fans and 64 sprinklers are installed beneath the ceiling in each barn (Fig. 4). The cooling system operates intermittently: the fans operate for 5 min and then sprinklers run for 1 min. The cow bedding is solid with rubber mattress or sandy soil. There are exercise areas outside the barns. Eleven barns housed a total of 1,450 heads of Holstein cows. Among them, 980 cows were lactating, with an average weight of 630 kg and an average daily milk production of approximately 27 kg. The cows were machine-milked three times daily at 4:00, 14:00 and 21:00. The remaining 470 cows were non-lactating, with an average weight of approximately 600 kg. All animals were fed three times daily at 8:30, 12:00 and 18:00 with total mixed rations. Manure was removed twice daily at 8:00 and 17:00 with a bulldozer. All experimental protocols used in this experiment were in accordance with those approved by the Northwest Agriculture and Forestry (A&F) University Institutional Animal Care and Use Committee (protocol number NWAFAC1022) and the institutional safety procedures were followed.

**Table 5:** Description of dairy barns.

Farm	Cow barns	Size (m) H × W × H	Housing density (m^2^/cow)	Bedding materials
Farm1	M1	72 × 31 × 7	12.2	Rubber cushion
Farm1	M2	72 × 31 × 7	11.7	Rubber cushion
Farm2	M3	72 × 26 × 6	12.9	Sandy soil
Farm2	M4	72 × 26 × 6	10.7	Sandy soil
Farm3	M5	96 × 27 × 7	10.1	Sandy soil
Farm3	M6	96 × 27 × 7	10.7	Sandy soil
Farm1	N1	72 × 31 × 7	13.6	Rubber cushion
Farm1	N2	72 × 31 × 7	13.2	Sandy soil
Farm2	N3	72 × 26 × 6	10.2	Sandy soil
Farm2	N4	72 × 26 × 6	10.2	Sandy soil
Farm3	N5	96 × 27 × 7	10.7	Sandy soil

Letter M means a lactating cow barn, while letter N indicates a non-lactating cow barn.

### Nitrogen and sulfur in feed and excretes

The dairy cows were fed a totally mixed ration (TMR). The average feed consumption was 44.5 kg/(AU·d) for lactating cows, while the average feed consumption was 41.5 kg/ (AU·d) for non-lactating cow. Fresh urine and fecal samples were collected three times daily (morning, noon and evening per day) for two days. The collected samples were stored at 4 °C before measurement on the same day. Nitrogen were determined by the Kjeldahl method. Sulfur were determined by the turbidimetric method^44^. The nitrogen and sulfur in feed and excreta are presented in Table 6.

**Table 6:** Nitrogen and Sulfur in the feed and their excreta by dairy cows.

Animal category	feed consumption (kg/(AU·d))	moisture content (%)	dry matter intake (kg/(AU·d))	feed N intake (kg/(AU·d))	excreted N (kg/(AU·d))	excreted N (%)	feed S intake (kg/(AU·d))	excreted S (kg/(AU·d))	excreted S (%)
Lactating cows	44.5	48.9	22.75	0.54	0.41	77.40	52.04	28.46	54.69
Non-Lactating cows	41.5	52.5	18.88	0.40	0.29	72.50	40.11	21.07	52.53

### Measurement of NH_3_ and H_2_S concentrations

**Figure 6:**
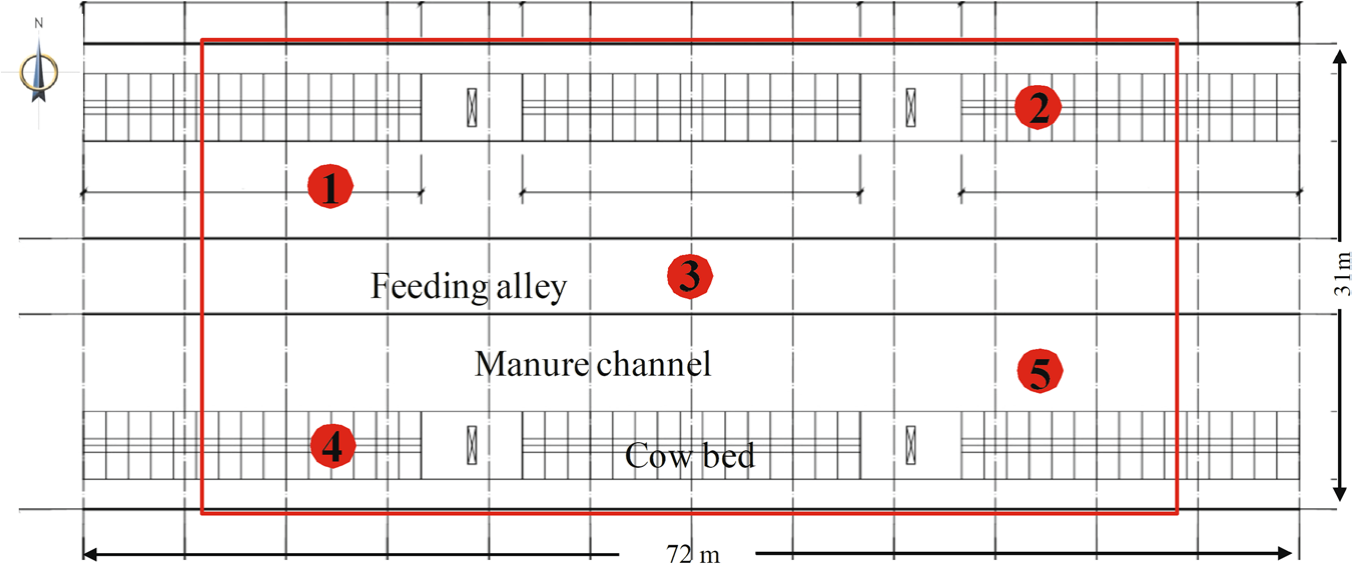
Sampling locations within a dairy barn. Locations 1 and 5 indicate manure channel, while locations 2 and 4 designate cow beds. Location 3 is the feeding aisle. Each location was sampled both near the floor and at 1.5 m above the floor.

NH_3_ and H_2_S concentrations were measured both inside and outside the barns. As shown in Figs 5 and 6 locations were sampled in each barn, including 2 manure areas, 2 cow bed locations and 1 feeding alley. Outside the dairy barns, sample locations included blank areas (upwind locations 20 m away from the barn), a manure storage area and a cow exercising area. Each location was sampled both near the floor and at 1.5 m above the floor. Near the floor is where NH_3_ and H_2_S gases are produced. The height at 1.5 m above the floor is approximately the breathing height for cows and dairy farmers. Gas samples were taken every two hours for 30 continuous minutes. The total sampling period was 48 h. The NH_3_ and H_2_S gases were collected using an integrated air sampler (2000C, Tuowei Instrument Ltd, Qingdao, China, flow range 0.1 L/min–1.0 L/min). A spectrophotometer (C752N754PC, Jinghua Instrument Ltd, Shanghai, China) was used for colorimetric analyses of NH_3_ and H_2_S concentrations. NH_3_ was measured using the Nessler’s reagent spectrophotometry method. NH_3_ in the air was absorbed using 0.05 mol/L dilute H_2_SO_4_. The generated NH_4_^+^ ions react with the Nessler reagent to form a yellow-brown complex. The absorbance of the complex proportional to the NH_3_ content was measured at a wavelength of 420 nm. The detection limit of NH_3_ was 0.01 mg/m^3^. Concentrations of NH_3_ were measured every 2 hours and calculated according to Eq. 3.

3 $${C}_{N{H}_{3}}\,or\,{C}_{{H}_{2}S}=(A-{A}_{0}-a)\times {V}_{s}\times D/(b\times {V}_{nd}\times {V}_{0})$$

${C}_{N{H}_{3}}$ or ${C}_{{H}_{2}S}$——NH_3_ or H_2_S concentration, mg/m^3^;

*A*——the absorbance of the sample;

*A*_0_——the absorbance of the blank with the same sample preparation liquid;

*a*——calibration curve intercept

*b*——calibration curve slope;

*Vs*——the total volume of sample absorption solution, mL;

*V*_0_——the volume of analyzed fluid, mL;

*V*_*nd*_——the standard volume of gas sample (101.325 kPa, 273 K), L;

*D*——dilution factor.

The H_2_S content was measured using the methylene blue spectrophotometric method^45^ with minor modifications. The absorbance was measured at a wavelength of 665 nm. The minimum detectable concentration was 0.001 mg/m^3^. Concentrations of H_2_S were measured every 2 hours and calculated according to Eq. 3.

### Environmental parameters

Environmental parameters were measured in the same locations and heights as for measurement of NH_3_ and H_2_S. The temperature, relative humidity (RH), wind speed, atmospheric pressure, CO_2_ and total suspended particles (TSP) inside and outside each barn were also measured. The temperature and humidity were recorded every 2 hours on an automatic temperature and humidity recorder (LGR-WSD20, Rogue Instrument Ltd, Hangzhou, China). Wind speed, atmospheric pressure, CO_2_ and TSP (Total suspended particles) were measured using an anemometer (405-V1, Testo, Lenzkirch, Germany), a barometer (DYM3 Yipin Instrument Ltd, Shanghai, China), a portable CO_2_ detector (JSA8, Jiada Instrument Ltd, Shenzhen, China) and a dust detector (JC-1000, Jingcheng Instrument Ltd, Qingdao, China), respectively.

### Calculation of ventilation rates

The ventilation rate is the rate at which air enters and leaves a building and is expressed in cubic meters per hour. The ventilation rate was calculated using two methods: CO_2_ balance method^46,47^, and the wind pressure and temperature difference forces method (WT method)^41^. The parameters required by the aforementioned methods were simultaneously measured in order to allow calculation of the ventilation rate at the same time, making comparison between the methods possible.

The emission rate of a gas was calculated using the following Eq. 4.

4 $${E}_{t}={Q}_{A}({C}_{i}-{C}_{o})$$

*E*_*t*_ — emission rate of a gas (g/h);

*Q*_*A*_ — adjusted ventilation rate (m^3^/h);

*C*_*i*_ and *C*_*o*_ —average concentrations (g/m^3^) of the gas inside and outside the building, respectively.

The weight of the cows and the production may differ from herd to herd. To make results comparable, the emission per animal unit (AU) was used in the modelling instead of emission per cow. The AU is equivalent to 500 kg animal mass^47^. The emission rate per AU can thus be stated as Eq. 5.

5 $$E={E}_{t}\times 500/(N\times m)\,$$

*E* — gas emission rate per animal unit (g/(AU·h))

*E*_*t*_ — the emission rate of a gas (g/h)

*N* —the total number of cows housed inside the building

*m* — the average mass of a cow accommodated in the building (kg/cow).

### Data analyses

Before statistical analysis, all data were checked and normalized if needed to satisfy the requirement of normality and homogeneity of variance. A mixed linear model was used to describe the effect of environmental and nutritional factors on NH_3_ or H_2_S concentrations as Eq. 6.

6 $${E}_{\begin{array}{c}ijk\end{array}}=\mu +{b}_{i}+{b}_{ij}+{\beta }_{1}N+{\beta }_{2}{T}_{in}+{\beta }_{3}N\cdot {T}_{in}+{e}_{ijk}$$

*E*_*ijk*_ was the dependent variable (NH_3_ or H_2_S concentration); *μ* is the overall mean of the dependent variable; bi was the barn, *i* = 1 to11, *b*_*ij*_ was the measuring height, *j* = 1,2; *β*_*1*_, *β*_2_, and *β*_3_ was the coefficient of fixed effect; *N∙T*_*in*_ represented the interaction between the N content in the feed and indoor temperature Tin; *e*_*ijk*_ represents random errors. All other environmental and nutritional factors and their interactions were also considered during the initial stage and were removed from the model due to insignificant effects.

The influence of independent variables (the N content in the feed *(N*_*f*_), N content in manure (*N*_*m*_), N content in the urine (*Nu*), S content in the feed (*S*_*f*_), S content of in manure (*S*_*m*_), S content in urine (*Su*), indoor temperature (*T*_*in*_), indoor wind speed *W, CO*_2_ and *TSP* concentrations) on NH_3_ and H_2_S concentrations inside the dairy barns (dependent variables) were first evaluated by calculating the Pearson’s correlation coefficient values. Then, the multi-collinearity among independent variables was examined by tolerance values and variance inflation factors. Finally, a multiple linear regression model was established by a stepwise procedure.

The fitting of the mixed linear model and the multiple linear regression method was performed using the SPSS 23.0 (IBM). Data were analyzed using one-way ANOVA with LSD multiple comparisons. The significance level was *P* < 0.05. Graphs were prepared using the Original Pro 8 software.
